# A feasibility study of provider-level implementation strategies to improve access to colorectal cancer screening for patients with schizophrenia: ACCESS2 (N-EQUITY 2104) trial

**DOI:** 10.1186/s43058-023-00541-0

**Published:** 2024-01-04

**Authors:** Masaki Fujiwara, Yuto Yamada, Tsuyoshi Etoh, Taichi Shimazu, Masafumi Kodama, Norihito Yamada, Takahiro Asada, Shigeo Horii, Takafumi Kobayashi, Teruo Hayashi, Yoshitaka Ehara, Kenji Fukuda, Keita Harada, Maiko Fujimori, Miyuki Odawara, Hirokazu Takahashi, Shiro Hinotsu, Naoki Nakaya, Yosuke Uchitomi, Masatoshi Inagaki

**Affiliations:** 1 Department of Neuropsychiatry, Okayama University Hospital, Okayama, Japan; 2 Department of Nursing, Shimane University Hospital, Izumo, Japan; 3 Division of Behavioral Sciences, National Cancer Center Institute for Cancer Control, National Cancer Center, Tokyo, Japan; 4 Okayama Psychiatric Medical Center, Okayama, Japan; 5 Zikei Hospital, Okayama, Japan; 6 Shimane Prefectural Psychiatric Medical Center, Izumo, Japan; 7 Seiwakai Nishikawa Hospital, Hamada, Japan; 8 Sekizen Hospital, Tsuyama, Japan; 9 Konan Hospital, Matsue, Japan; 10 Department of Gastroenterology, Okayama Saiseikai General Hospital, Okayama, Japan; 11 Division of Survivorship Research, National Cancer Center Institute for Cancer Control, National Cancer Center, Tokyo, Japan; 12 Division of Screening Assessment and Management, National Cancer Center Institute for Cancer Control, National Cancer Center, Tokyo, Japan; 13 Department of Biostatistics and Data Management, Sapporo Medical University, Sapporo, Japan; 14 Tohoku Medical Megabank Organization, Tohoku University, Sendai, Japan; 15 Department of Psychiatry, Faculty of Medicine, Shimane University, Izumo, Shimane 693-8501 Japan

**Keywords:** Cancer screening, Schizophrenia, Case management, Provider level, Implementation

## Abstract

**Background:**

People with schizophrenia have a lower colorectal screening rate than the general population. A previous study reported an intervention using case management to encourage colorectal cancer screening for patients with schizophrenia in psychiatric outpatient settings. In this feasibility study, we developed provider-level implementation strategies and evaluated the feasibility of conducting a definitive trial in terms of the penetration of the intervention assessed at the patient level. Additionally, we examined the fidelity of strategies to implement the intervention at the provider level in a routine clinical psychiatric setting.

**Methods:**

This was a multicenter, single-arm feasibility study with medical staff at psychiatric hospitals in Japan. The provider-level implementation strategies developed in this study included three key elements (organizing an implementation team appointed by the facility director, interactive assistance using a clear guide that outlines who in the hospital should do what, and developing accessible educational materials) to overcome major barriers to implementation of the intervention and four additional elements (progress monitoring, joint meetings and information sharing among participating sites, adaptation of encouragement methods to specific contexts, and education of on-site staff), with reference to the elements identified in the Expert Recommendations for Implementing Change (ERIC). The feasibility of the strategies was evaluated by the proportion of patients who were eligible for encouragement (patients with schizophrenia aged 40, 50, or 60) who received encouragement. We set the goal of providing encouragement to at least 40% of eligible patients at each site.

**Results:**

Two public and four private psychiatric hospitals in Okayama and Shimane prefectures participated in this study. Regarding fidelity, all elements of the strategies were conducted as planned. Following the procedures in the guide, each team prepared and provided encouragement according to their own facility and region. Penetration, defined as the proportion of eligible patients who received encouragement, ranged from 33.3 to 100%; five of the six facilities achieved the target proportion.

**Conclusions:**

The provider-level implementation strategies to implement encouragement were feasible in terms of penetration of the intervention assessed at the patient level. The results support the feasibility of proceeding with a future definitive trial of these strategies.

**Trial registration:**

jRCT, jRCT1060220026. Registered on 06/04/2022.

**Supplementary Information:**

The online version contains supplementary material available at 10.1186/s43058-023-00541-0.

Contributions to the literature

- This was a feasibility study of a provider-level implementation strategy to implement an evidence-based intervention to encourage colorectal cancer screening for patients with schizophrenia in a psychiatric outpatient setting.
- Key elements of the strategy are as follows: (i) organizing an implementation team appointed by the facility director, (ii) creating a clear guide that outlines who should do what, and (iii) developing accessible educational materials.
- The current findings may be helpful for implementing evidence-based interventions to improve cancer screening disparity among patients with schizophrenia, and the implementation strategies may be applicable to other psychosocial interventions in settings with a similar context.

## Background

Schizophrenia is considered to be one of the most serious mental illnesses. People with schizophrenia have a life expectancy that is 13–15 years shorter than that of the general population [1]. Reducing this health disparity is a global challenge. Cancer is a leading cause of death in people with schizophrenia, who have higher cancer mortality rates than the general population [2, 3]. Delayed diagnosis of cancer has been suggested to be one of the factors contributing to high cancer mortality rate in this population [4].

Cancer screening, which has been shown to be effective in the early detection of cancer and reduction of cancer mortality, is one of the basic components of cancer control [5]. However, people with schizophrenia have lower cancer screening rates than the general population [6–8]. Therefore, it is necessary to establish a strategy to deliver cancer screening to people with schizophrenia. Among various types of health screening, cancer screening has a high level of evidence of effectiveness [9–11], and cancer has a severe disease burden [12]. Therefore, implementing cancer screening is considered to be a high priority [13].

A Cochrane review by Barley et al. reported that there were no randomized controlled trials of interventions designed to encourage uptake of cancer screening among people with severe mental illness [14]. To the best of our knowledge, only two single-arm trials have been conducted to examine interventions to encourage breast cancer screening [15] and lung cancer screening [16] for people with severe mental illness. Thus, there is an urgent need to establish evidence for interventions to encourage cancer screening among people with schizophrenia and to implement effective interventions in routine health care.

Irwin et al. suggested that patient-, provider-, and systems-level factors contribute to low cancer screening rates among people with schizophrenia [17]. At the patient level, symptom severity is reported to be associated with nonreceipt of cancer screenings, suggesting the need to support patients with schizophrenia on the basis of their impairments [6]. At the systems level, in Japan, cancer screening is not offered at psychiatric outpatient clinics. In a previous study, patients with schizophrenia who did not have current visits to outpatient clinics for any physical illnesses were found to be less likely to receive cancer screening than those who did [6]. Therefore, psychiatric providers are expected to encourage patients with schizophrenia to undergo cancer screening [6, 17].

On the basis of the context identified in our previous observational studies investigating cancer screening uptake among patients with schizophrenia in Japan [6, 18], we developed an intervention using case management to encourage patients with schizophrenia to undergo colorectal cancer (CRC) screening in psychiatric outpatient settings [19]. In our proposed intervention, medical staff provide education and navigation of CRC screening to patients with schizophrenia [19]. We tested the intervention in a randomized controlled trial, revealing that the intervention increased CRC screening uptake among patients with schizophrenia compared with usual care [20].

For medical staff to provide the intervention described above in routine clinical psychiatric settings, it is necessary to establish provider-level implementation strategies. A manual for providing encouragement for CRC screening was developed in the previous randomized controlled trial [20]. In our previous study (the ACCESS study) [20], providers were involved as the research team because the intervention study was designed to change patient behavior in a clinical trial setting. However, provider-level implementation strategies for offering the intervention in routine practice have not been developed. Additionally, a qualitative study conducted in conjunction with a previous randomized controlled trial indicated challenges in implementing the intervention in routine clinical settings [21]. Adaptation of the intervention to increase its feasibility in routine clinical settings is also needed.

To provide encouragement to patients with schizophrenia to engage in CRC screening under routine clinical settings, we developed provider-level implementation strategies and adapted the intervention. A recent review noted incomplete specification of implementation strategies in the mental health literature [22]. Therefore, we incorporated elements of implementation strategies from an existing pool of implementation strategies identified in the Expert Recommendations for Implementing Change (ERIC) [23] and defined their specifications to increase replicability and develop a useful set of implementation strategies. To the best of our knowledge, no previous studies have reported provider-level implementation strategies for interventions to encourage cancer screening uptake for people with severe mental illness. However, several review articles have examined studies reporting implementation strategies for psychosocial interventions for people with severe mental illness in psychiatric settings [24, 25].

This feasibility study addressed several areas of uncertainty [26–28] about a future definitive trial with the primary endpoint of CRC screening uptake among patients with schizophrenia. Whether psychiatric hospitals adopting the strategies will actually be able to provide encouragement to their patients with schizophrenia is currently uncertain and should be evaluated. Therefore, in this feasibility study, we first evaluated the penetration of the intervention assessed at the patient level (i.e., the proportion of eligible patients who received encouragement via the provider-level implementation strategies). Second, we qualitatively described the fidelity of the implementation strategies (i.e., the way in which each element of the strategies was conducted in accordance with the planned specifications).

## Methods

### Trial design

This was a multicenter, single-arm trial with the medical staff of a psychiatric hospital. The study was approved by the institutional ethics committee of Okayama University. This study was registered in the Japan Registry of Clinical Trials (ID: jRCT1060220026).

### Setting (cancer screening in Japan)

In Japan, the Ministry of Health, Labour and Welfare recommends the following five cancer screenings: annual fecal occult blood test (FOBT) for CRC screening and chest X-ray for lung cancer screening for people aged 40 and older, biannual upper gastrointestinal X-ray or upper endoscopy for gastric cancer screening for people aged 50 and older, biannual mammography for breast cancer screening for people aged 40 and older, and biannual Papanicolaou (PAP) smear test for people aged 20 years and older [29].

In Japan, local governments provide population-based cancer screening. Municipal cancer screening programs cover unemployed people, employees of small- to medium-sized companies, and self-employed individuals. For people with severe mental illness, municipal cancer screening programs are the primary opportunity to undergo cancer screenings.

Municipal cancer screenings are not provided year-round, and each municipality has a specific period during which the screenings are provided. Although the content of the cancer screening tests is similar in all municipalities, there are differences among municipalities in the way the tests are provided. For example, in some municipalities, residents receive the FOBT kit at the clinic and bring it to the clinic, whereas in other municipalities, residents receive the FOBT kit by mail and submit it by mail. Additionally, some municipalities provide free coupons, whereas others do not. Furthermore, the procedures for obtaining free coupons vary between municipalities.

### Participants

This study included two public and four convenient private psychiatric hospitals in Okayama and Shimane prefectures. The Department of Psychiatry at Okayama University and Shimane University undertake ongoing clinical and educational collaborations with a number of psychiatric hospitals, including the six hospitals included in this study. Hospitals of different sizes and in different cities were included to assess the feasibility of provider-level implementation strategies. We explained the current study to the hospital directors, who agreed with the importance of encouraging cancer screening, and obtained their agreement to participate in the study. All of these facilities are standard psychiatric hospitals in Japan and do not provide cancer screening tests. Consent to participate in the study was obtained from the director of each hospital. Staff members participating in a team to implement the encouragement of CRC screening at each hospital were targeted for the research intervention, and consent was obtained from individual staff members.

### Provider-level implementation strategies

In developing the provider-level implementation strategies used in this study, researchers (M. F.^1^, Y. Y., M. I.; psychiatrists) and medical staff who provided encouragement to engage in CRC screening in a prior study discussed the barriers to providing encouragement in routine practice. This discussion suggested the following major barriers to implementation of encouragement: (i) staff engagement with encouragement, (ii) the degree to which methods of behavior and tasks for conducting the encouragement are developed in advance, and (iii) access to knowledge and information. A strategy development team composed of the researchers mentioned above and an implementation science expert (TS) discussed and identified the following elements as key strategies to overcome these barriers: (a) organizing an implementation team appointed by the facility director, (b) interactive assistance using a clear guide that outlines who in the hospital should do what, and (c) developing educational materials that are accessible to busy medical staff. During this discussion, the team considered findings regarding implementation strategies presented in previous studies to promote the uptake of evidence-based psychosocial interventions for people with severe mental illness [24]. In addition, the strategy development team discussed and identified elements of the implementation strategy identified in ERIC [23] that were considered to be useful and feasible in this setting. The following four elements were also incorporated to develop a set of provider-level implementation strategies in the current study: (d) progress monitoring, (e) joint meetings and information sharing among participating sites, (f) adaptation of the encouraging methods to the specific context, and (g) education of on-site medical staff. The specification of the implementation strategies was predefined with actor, action, target of actions, temporality, implementation outcome affected, and justifications, in accord with Proctor’s Reporting Framework domains (shown in Table 1) [30]. Depending on the element of the strategy, the actors could be the researchers or the implementation team at each hospital.

**Table 1:** Provider-level implementation strategies

Elements of the strategies used in this study (ERIC classification)	Domain					
	**Action(s)**	**Target(s)**	**Temporality**	**Dose**	**Outcomes affected**	**Justification**
Organizing an implementation team appointed by the facility director (developing stakeholder interrelationships)	The researchers explain the implementation team to the facility director	Appointment of implementation leaders by the facility director	Phase of pre-adoption or preparation	Once (approximately 30 min)	Fidelity and feasibility	Engaging (CFIR); formally appointed internal implementation leaders (CFIR)
Interactive assistance using a clear guide that outlines who in the hospital should do what (providing interactive assistance)	Researchers explain the guide for encouraging cancer screening at psychiatric institutions	The facility director’s knowledge of cancer screening and encouragement methods and their judgment about the adoption of the encouragement method	Phase of pre-adoption	Once (approximately 30 min)	Acceptability, adoption (i.e., research participation)	Readiness for implementation (CFIR)
	Researchers explain the guide. After that, regular meetings and occasional email consultations are provided	Preparation and implementation of the encouragement of cancer screening by the implementation team	Phase of preparation and implementation of encouragement	(1) Startup meeting (60 min); (2) regular 15-min meetings twice a month for the first 2 months; (3) regular meetings once a month thereafter. E-mail consultation as needed	Acceptability, adoption, fidelity, and feasibility	Knowledge and beliefs about the intervention (CFIR) and access to knowledge and information (CFIR)
Progress monitoring (using evaluative and iterative strategies)	The researchers monitor progress at each site according to the items in the guide through regular meetings	Implementation team’s ability to lead and manage the implementation of encouragement	Phase of preparation and implementation of encouragement	(2) Regular 15-min meetings twice a month for the first 2 months; (3) regular meetings once a month thereafter	Fidelity and feasibility	Reflecting & evaluating (CFIR)
Joint meetings and information sharing among participating sites (developing stakeholder interrelationships)	The researchers share the efforts of the implementation team at each hospital through regular meetings	Preparation and implementation of encouragement by the implementation team	Phase of preparation and implementation of encouragement	(2) Regular 15-min meetings twice a month for the first 2 months; (3) regular meetings once a month thereafter. E-mail consultation as needed	Feasibility	Cosmopolitanism (CFIR)
Adaptation of the encouraging methods to the specific context (adapting and tailoring to context)	Researchers adapt the methods of encouraging cancer screening using case management to the context (modification of the methods to consider daily clinical resources, modification of the materials)	The methods of encouraging colorectal cancer screening using case management	Phase of pre-adoption (adaptation will be made in subsequent phases if necessary)	As needed	Adoption, acceptability, fidelity, and feasibility	Adaptability (CFIR); cost (CFIR); Fujiwara et al. (2021) and Yamada et al. (2022)
	The implementation team adapts the materials to suit the region. In addition, a method of implementing encouragement is established for each facility	The methods of encouraging colorectal cancer screening using case management	Phase of preparation and implementation of encouragement	As needed	Acceptability, fidelity, and feasibility	Adaptability (CFIR); Fujiwara et al. (2021) and Yamada et al. (2022)
Developing educational materials that are accessible to busy medical staff (training and educating stakeholders)	Researchers distribute educational materials via the website and provide educational videos	Implementation team’s knowledge of how to implement encouragement of colorectal cancer screening	Phase of preparation and implementation of encouragement	Presentation of videos for 35 min in total (nine videos)	Acceptability, fidelity, and feasibility	Knowledge and beliefs about the intervention (CFIR)
Education of on-site medical staff (training and educating stakeholders)	The implementation team educates the medical staff about the methods of encouragement and its implementation in the facility	On-site medical staff’s knowledge of how to implement encouragement of colorectal cancer screening	Phase of preparation and implementation of encouragement	Presentation of videos for 35 min in total (nine videos) and on-site meetings (as many times as needed)	Acceptability, fidelity, and feasibility	Knowledge and beliefs about the intervention (CFIR)

*ERIC* Expert Recommendations for Implementing Change *CFIR* Consolidated Framework for Implementation Research

#### A guide for encouraging cancer screening at psychiatric institutions

A guide was developed that outlines what facility directors and implementation teams should do to implement the encouragement of cancer screening at each facility (Supporting file; a guide for encouraging cancer screenings at psychiatric institutions in English and Japanese). This guide describes the process in four steps: (i) the director organizes a team to implement the encouragement of cancer screening, (ii) the implementation team prepares the encouragement in accordance with the guide, (iii) the implementation team educate staff, and (iv) staff provide encouragement for patients to engage in CRC screening using case management.

#### Development and distribution of educational materials

Educational materials for learning about cancer screening and a method of encouragement using case management were developed. The materials consisted of the following: (i) an overview of cancer screening, (ii) cancer screening provided by municipalities, and (iii) the method of encouragement using case management. Nine videos of 3–5 min in length were made available on our research website for staff at each facility to view at any time. Each of the materials was also made available as a downloadable pdf file.

### Intervention using case management to encourage colorectal cancer screening

The method of cancer screening encouragement using case management has been described in detail in previous papers [19, 20]. The elements of the intervention were described according to the Template for Intervention Description and Replication (TIDieR) (shown in Supporting Table 1) [31]. Case managers (nurses or mental health social workers working in the outpatient department of each hospital) provide counseling sessions for eligible patients. This intervention aimed to educate and guide patients regarding CRC screening. The first session includes the following three components: (i) education for CRC screening, (ii) assistance in making decisions and an appointment for CRC screening, and (iii) assistance in obtaining a coupon for free screening. Follow-up sessions are generally scheduled in conjunction with outpatient visits after an initial session but may be skipped on the basis of the clinical judgment of the case manager.

In the current study, the intervention used in a previous study was adapted to increase its feasibility in routine clinical settings. Our previous study suggested that providing encouragement using case management for all patients with schizophrenia over 40 years of age in a single year under routine practice would be difficult in terms of human resources [21]. Therefore, after preliminary interviews with participating facilities, the strategy was adapted to provide encouragement to patients with schizophrenia who were aged 40, 50, or 60 years old each year. Patients of other ages would be reminded of the screening with a simple flyer. The goal was to continue this implementation strategy annually over time to provide encouragement to all target patients. In addition, the strategy allows each facility to adjust the extent to which it offers encouragement on the basis of the number of eligible patients and the facility’s resources. For example, some hospitals could offer encouragement to patients every 5 years of age (i.e., at 40, 45, 50, 55, or 60 years old). By starting with the number of eligible patients according to each facility’s resources, the feasibility of offering encouragement can be increased.

### Data collection and outcome measures

#### Primary outcome

As a measure of the penetration of the intervention using case management to encourage CRC screening at the patient level, the proportion of patients who were eligible for the intervention (patients with schizophrenia aged 40, 50, or 60 years old) who received at least one session for encouragement was calculated. Whether or not patients received encouragement was determined retrospectively from their medical records. If encouragement was not provided, the reason was recorded. The guide required providers to document whether they provided encouragement in each patient’s medical record. The denominator for calculating this proportion was the number of patients with schizophrenia aged 40, 50, or 60 years old with at least two visits to the facility between June and December 2022. This definition was chosen because encouragement was provided to patients who continued to visit each facility as their primary psychiatric clinic.

The present feasibility study sets a goal of providing encouragement to at least 40% of eligible patients at each site, which constituted the study’s progression criteria [27, 28]. On the basis of our previous ACCESS study [20], we estimated that 15% of eligible patients were deemed by their primary psychiatrists to be inappropriate for encouragement because of their medical condition, 20% could not be contacted because of the timing of patient visits or because the outpatient departments were overly busy, and 40% of eligible patients refused or omitted the encouragement. Thus, we estimated that 0.85 × 0.8 × 0.6 × 100 = 40.8% of eligible patients would receive encouragement. If the penetration of the intervention in this study was similar to that in the previous ACCESS study conducted under ideal settings, we considered that we could proceed to a definitive trial.

#### Secondary outcomes

The way in which each element of the provider-level implementation strategies was conducted was described as the fidelity assessment of the implementation strategies of this study. These descriptions were based on transcripts of meetings between the researchers and the implementation teams, as well as interviews with the implementation teams after the encouragement ended.

### Variables

The following information about the participating facilities was obtained from the directors: number of beds, number of psychiatrists for outpatient services, number of non-physician staff for outpatient services, average number of outpatients per day from June to December in 2022, and number of patients with schizophrenia aged 40, 50, or 60 years old who were targeted for providing encouragement in the study.

### Data analysis

#### Primary outcome

For each of the participating facilities, the number of patients who were eligible for encouragement (outpatients with schizophrenia aged 40, 50, or 60 years old) and the number of patients who received encouragement were reported. The proportion of eligible patients who received encouragement was calculated.

#### Secondary outcomes

Two researchers (M. F.^1^ and Y. Y.) discussed and summarized how each element of the provider-level implementation strategies was conducted on the basis of meeting records and interviews with the implementation team. For each element, these summaries were qualitatively described.

## Results

### Background data for participating facilities

Background data for the participating facilities are shown in Table 2. Three facilities from Okayama Prefecture (Hosp. 1–3) and three from Shimane Prefecture (Hosp. 4–6) participated; Hosp. 1 and Hosp. 4 are public psychiatric hospitals and are the core hospitals for psychiatric care in the prefecture. The other private hospitals also provide both outpatient and inpatient psychiatric care to residents of the surrounding municipalities.

**Table 2:** Background data for participating facilities

	Hosp. 1	Hosp. 2	Hosp. 3	Hosp. 4	Hosp. 5	Hosp. 6
City	Okayama	Okayama	Tsuyama	Izumo	Hamada	Matsue
Number of beds	255	570	295	224	402	147
Number of psychiatrists for outpatient services	32	24	11	11	9	4
Number of nonphysician staff for outpatient services	21	11	16	18	15	4
Average number of outpatients per day	126	156	104	101	95	27

### Primary outcome: proportion of eligible patients who received encouragement using case management

Table 3 shows the number of patients who were eligible for encouragement and the proportion of eligible patients who received the encouragement for each facility. The number of patients ranged from 2 at the facility with the lowest number to 67 at the facility with the highest number. The percentage of patients who received encouragement ranged from 33.3 to 100%. Five of the six facilities achieved a proportion of more than 40%.

**Table 3:** Proportion of eligible patients who received encouragement using case management

	Hosp. 1	Hosp. 2	Hosp. 3	Hosp. 4	Hosp. 5	Hosp. 6
Number of eligible patients for encouragement (outpatients with schizophrenia aged 40, 50, or 60 years old)^a^	61	67	27	20	8	2
Number of eligible patients for whom the encouragement was omitted^b^	10	7	2	0	1	0
Number of eligible patients for whom the encouragement was deemed inappropriate by their psychiatrists	4	6	1	1	0	0
Number of eligible patients who received encouragement^c^	24	20	18	10	4	2
Number of eligible patients who declined to receive encouragement	15	21	3	0	3	0
Number of eligible patients who could not be contacted	8	13	3	9	0	0
Proportion of eligible patients who received encouragement^d^	47.0%	33.3%	72.0%	50.0%	57.1%	100.0%

^abcd^*d* = c/(a − b) × 100 shows how to calculate the Proportion of eligible patients who received encouragement

### Secondary outcomes: description of how the provider-level implementation strategies were conducted

Table 4 shows how each element of the strategies was conducted. All elements were conducted in accordance with the specifications. Implementation teams were organized by facility directors at all participating facilities. Following the procedures in the guide, each team prepared and provided encouragement according to their own facility and region.

**Table 4:** Description of how the provider-level implementation strategies were conducted

Elements of the implementation strategies	How the provider-level implementation strategies were conducted
Organizing an implementation team appointed by the facility director	Implementation teams were organized at all facilities. In each facility, the team was composed of members according to the internal conditions of the facility
Interactive assistance using a clear guide that outlines who in the hospital should do what	The directors of the six facilities agreed to participate in the study after receiving explanations of the guide from the researchers as per the specifications
	All implementation teams attended the startup meeting as specified and were briefed by the researcher in the guide. The teams then attended regular meetings; many teams were too busy to attend regular meetings because of the COVID-19 pandemic. The implementation teams communicated with the researchers via email on a regular basis, or as needed
Progress monitoring	The implementation teams prepared for the provision of the encouragement according to the guide. The research team received progress reports from the implementation teams in regular meetings or via email
Joint meetings and information sharing among participating sites	Through the meetings, the progress of the participating facilities was shared. In addition, helpful materials developed at participating sites were shared with the other sites
Adaptation of the encouraging methods to the specific context	Researchers adopted the strategy to begin providing encouragement on a smaller scale, depending on the resources of each facility. See “Methods” section patient-level implementation strategies for details
	The implementation teams made minor modifications to the materials used for the encouragement to suit the region. The teams determined the range of patients who would be eligible for the encouragement on the basis of their estimates of patient volume. Depending on the facility’s outpatient system, a flow for providing encouragement was developed
Developing educational materials that are accessible to busy medical staff	The implementation teams were able to access educational materials from the study website as per specifications. The implementation teams did not require any additional educational materials other than those prepared by the researcher
Education of on-site medical staff	The implementation teams held a meeting at the facility to inform the on-site medical staff about the study and the flow for providing encouragement. The teams used educational materials to educate on-site medical staff. At some sites, members of the implementation team provided simulated encouragement as a demonstration

## Discussion

This study developed provider-level implementation strategies to implement evidence-based interventions to encourage CRC screening in patients with schizophrenia in a routine psychiatric clinical setting. The results showed that the elements of the provider-level implementation strategies were conducted according to the pre-defined implementation strategies, and all participating sites provided the CRC screening encouragement using case management according to the guide. To the best of our knowledge, no previous studies have reported provider-level implementation strategies for encouraging cancer screening uptake for patients with severe mental disorders on the basis of implementation science. An important strength of the current study is that we developed a set of strategies by combining existing implementation strategies and detailed their specifications using implementation strategy taxonomies.

The provider-level implementation strategies in this study enabled five of the six facilities to achieve the target proportion of encouragement provision. In addition, the fidelity of the implementation strategies was good, as shown qualitatively in Table 4. One facility did not reach the target proportion. This facility had a higher number of outpatients per day compared with other facilities, and relatively fewer outpatient staff resources, which might have been related to this result. During progress monitoring, the implementation team in this facility reported that outpatient staff resources were scarce because of work related to the COVID-19 pandemic, and it was difficult to secure staff to provide encouragement for CRC screening. This provider-level implementation strategy might show even better feasibility if the human resource burden caused by the COVID-19 pandemic is reduced.

A number of previous studies have reported implementation strategies for psychosocial interventions for people with severe mental illness in psychiatric settings, although the content of these interventions differs from that in the present study. Islam et al. reported a systematic review of factors affecting the implementation of supported self-management interventions for people with severe mental health problems in secondary mental health care settings [25]. Menear et al. reported a review of implementation strategies to promote the uptake of evidence-based psychosocial interventions for people with severe mental illness [24]. In the current study, we developed provider-level implementation strategies for implementing a psychosocial intervention to encourage CRC screening in psychiatric settings. Therefore, there are many points that can be compared with the findings of implementation strategies obtained in the previous studies mentioned above.

The use of the strategy of organizing an implementation team appointed by the facility director, which was the first of the key strategies in this study, appeared to be useful. In two previous reviews [24, 25], identifying and appointing a champion to manage the implementation of the interventions and organizing an implementation team were also identified as facilitators of the interventions. A review by Islam et al. reported that senior leadership is important, and that a lack of senior support can be a barrier [25], particularly when there are competing demands for resources [32]. In the current study, no staff were hired to provide encouragement for CRC screening, and existing resources in the outpatient setting were used to provide encouragement. Thus, the provision of encouragement may be in competition with other duties. The positioning of the implementation team’s activities under the leadership of the facility director would have allowed for encouragement to be implemented at all participating facilities under such circumstances. In addition, the fact that the implementation team was composed of members from multiple professions who were able to lead the hospital’s stakeholders was considered a facilitating factor for implementation in the current study. Islam et al.’s review reported that the lack of involvement of multidisciplinary staff was a barrier to the implementation of interventions [25]. The current findings reaffirm the importance of support from senior leadership and multidisciplinary involvement in organizing an implementation team.

The second of the key strategies, interactive assistance using a clear guide that outlines who in the hospital should do what, was also considered effective. The extent to which the plans and methods of behavior and tasks for implementing interventions are prepared in advance is relevant to facilitating effective implementation [33]. In the present study, the implementation team at each facility followed the guide and reviewed the implementation process tailored to their site and was able to educate on-site medical staff and provide encouragement to engage in CRC screening. The guide includes the third of the key strategies: the provision of educational materials that are accessible to busy medical staff. In two previous reviews [24, 25], educational strategies, including the distribution of educational materials, training, and supervision, were identified as a core strategy or facilitator in implementation studies of psychosocial interventions. The encouragement of cancer screening is within the scope of support provided in daily clinical practice. Therefore, as in our previous study, the implementation teams and medical staff at each site were able to learn about the method of encouragement and deliver it using the educational materials provided with the guide, without any special training provided by outside organizations. In terms of feasibility, it is important to develop educational strategies that are minimally burdensome and accessible to providers, taking into account the nature of the intervention, the skills of the providers, and the settings.

In the current study, the intervention was adapted prior to implementation to improve the feasibility under routine clinical settings. Two previous review articles reported that adaptability of the intervention and the ability to take a flexible approach were important features for improving implementation [24, 25]. As described in the “Methods” section, we adapted the intervention to provide encouragement using case management for a small number of eligible patients on the basis of the number of patients at each site. When an intervention is complex, taking an incremental approach to implementing the intervention increases the likelihood of success [33]. All participating hospitals were particularly busy during the period of this study because of the COVID-19 pandemic. Therefore, implementation of encouragement was likely to place a heavy burden on routine medical care. Operating the encouragement flexibly and gradually expanding the provision were considered a useful strategy for the implementation team.

### Implications

In a series of studies conducted in our research group, including the current study, we developed an effective intervention to encourage patients with schizophrenia to undergo CRC screening in psychiatric outpatient settings [20], as well as outlining provider-level implementation strategies to implement the intervention in routine care settings. The elements required for direct support for patients may be common across health systems, although this intervention to encourage CRC screening was developed in Japan. The intervention using case management and provider-level implementation strategies might be applicable in similar settings, where there are healthcare providers who provide ongoing support to patients with schizophrenia. Additionally, the intervention and implementation strategies were developed for people with mental disorders and could potentially be adapted to encourage cancer screening for people with intellectual disabilities.

Importantly, the proportion of patients who received encouragement varied widely among the participating facilities. The results indicated that the proportions were low in relatively busy facilities with high outpatient volumes. Modifying the implementation strategies to improve the percentage of encouragement provided at such facilities is a challenge for future studies. It may be useful for each facility to consider extending the period for which encouragement is offered, depending on the progress of encouragement.

In addition, to scale up encouragement to engage in CRC screening in accordance with the guide developed in this study, it will be necessary to develop implementation strategies to encourage psychiatric facilities to adopt this approach on a regional or national basis. To this end, it may be desirable to incorporate external settings that promote the implementation of encouragement as multi-level implementation strategies.

### Limitations

First, participating facilities were able to provide encouragement using cases in a single year, but it is not known whether this encouragement could be provided on an ongoing basis in subsequent years. This study is ongoing, and results will be obtained in the future regarding the sustainability of providing encouragement. Second, the costs and economic outcomes of implementing the encouragement in this study were not evaluated, although the intervention was developed to be implemented within current clinical settings without additional resources. Our previous ACCESS study revealed the average time required to provide encouragement. Third, it is not known whether patients who received encouragement to engage in CRC screening in this study were screened for CRC. Because individual consent from the patient is required to obtain information regarding the patient’s CRC screening participation in a clinical study, the evaluation of participation was omitted in this study for pragmatic reasons. Fourth, receipt of the encouragement was determined retrospectively from the medical records. Thus, the accuracy with which the providers documented the encouragement was unclear. Fifth, at some participating hospitals, the number of patients who were eligible for the encouragement was very small, which could lead to overestimation of the proportion of eligible patients who received encouragement.

## Conclusion

The current results indicated that the provider-level implementation strategies developed in this study to implement encouragement using case management for CRC screening among patients with schizophrenia were feasible in terms of penetration of the intervention assessed at the patient level. These findings support the feasibility of proceeding with a future definitive trial of the strategies. Although the strategies appeared to be feasible, the proportion of patients who received encouragement varied widely among participating facilities. To increase this proportion, it may be desirable to consider possible modifications in the implementation strategies.

### Supplementary Information

**Additional file 1: Supporting file.** A guide for encouraging cancer screening at psychiatric institutions.

**Additional file 2: Supporting Table 1.** Intervention using case management to encourage CRC screening.

## Data Availability

An anonymous analyzed data set will be made available to researchers upon reasonable request to the corresponding author.

## Abbreviations

CRC: Colorectal cancer
ERIC: Expert Recommendations for Implementing Change
FOBT: Fecal occult blood test
