# Supplementary material for: A feasibility study of provider-level implementation strategies to improve access to colorectal cancer screening for patients with schizophrenia: ACCESS2 (N-EQUITY 2104) trial
Source: Implement Sci Commun. 2024 Jan 4;5:2. doi: 10.1186/s43058-023-00541-0 (PMC10768128; doi:10.1186/s43058-023-00541-0)
Supplement: Supplementary file 1 — Additional file 1: Supporting file. A guide for encouraging cancer screening at psychiatric institutions. [file 43058_2023_541_MOESM1_ESM.docx]

**A guide for encouraging cancer screening**  **at psychiatric institutions**

A Feasibility Study of Case Management by Psychiatric Care Providers to Improve Access to Cancer Screening for Patients with Schizophrenia in Usual Clinical Practice

October 10, 2021 Version 1.0

In psychiatric care, we often encounter schizophrenic patients who are found to have advanced cancer and are unable to receive standard treatment such as surgery or anticancer drugs. Cancer screening is effective for the early detection and treatment of cancer. In Japan, it is recommended that individuals undergo five cancer screenings, and municipalities provide cancer screening services. However, there are disparities in screening rates for people with mental disorders, with a rate of approximately one-half that of the general population.

If individual encouragement to engage in cancer screening is performed by staff of psychiatric institutions in accordance with this guide, the colorectal cancer screening rate among patients with schizophrenia, who are known to have the lowest uptake rate, could potentially increase to the same level as that of the general population. Many patients are concerned about cancer screening but do not participate in it because they do not understand it well. Some of our patients reported that explanations and guidance for cancer screenings from outpatient staff were very helpful.

We would appreciate your cooperation in improving the disparities in cancer screening uptake and cancer mortality rates among people with mental disorders.

Principal Investigator: Masaki Fujiwara (Okayama University)

Research Group Representative: Masatoshi Inagaki (Shimane University)

**The following steps should be taken to prepare and implement the encouragement of cancer screening at your facility.**

**1. The director organizes a team to implement the encouragement of cancer screening**

**2. The team prepares the encouragement of cancer screening**

**3. The team educates staff involved in encouraging cancer screening**

**4. Staff provide encouragement for patients to engage in cancer screening**

**1．Organizing a team to implement the encouragement of cancer screening**

- Compose a multidisciplinary implementation team. The implementation team should be a team of psychiatrists involved in the outpatient department, as well as nurses or mental health social workers.
- This team will lead the encouragement of cancer screening according to the procedures outlined in this guide.
- The implementation team should include psychiatrists, nurses, and mental health social workers who are familiar with the outpatient department at your facility.
- The implementation team and its members will be known throughout your facility.

**
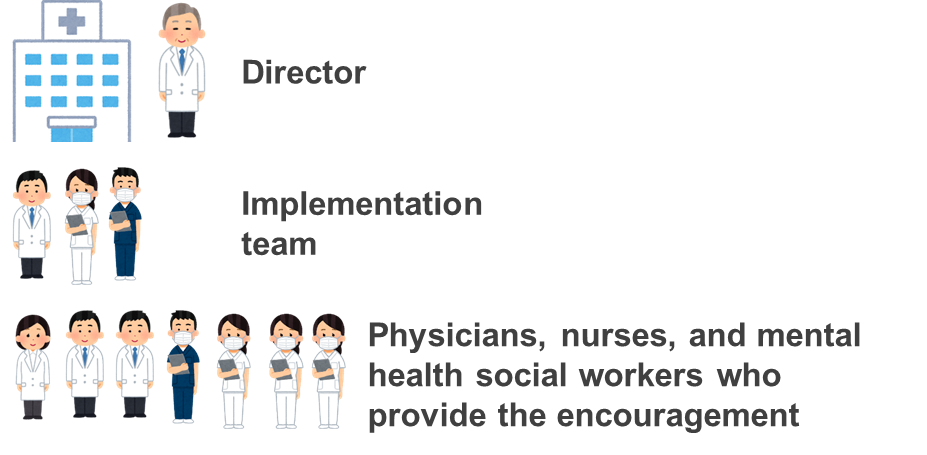
**

**２．Preparing for the encouragement of cancer screening**

- ***2.1 Team members gain an understanding of cancer screening and the method of encouragement***
  - Understanding general knowledge of the contents of the major cancer screenings (Educational Resource 1)
  - Understanding the system of municipal cancer screening and the procedures for receiving screening (Educational Resource 2)
  - Understanding the key points of the methods for encouraging cancer screening using case management (Educational Resource 3)
- ***2.2 Review the cancer screening programs in the municipalities surrounding your facility and modify materials for encouragement to use at your facility.***
  - Brochures explaining the cancer screening program are available on each municipality’s website.
- ***2.3*** ***Decide how to provide encouragement of cancer screening.***
  - Decide how to operate so that psychiatrists do not miss eligible patients for encouragement (the first goal is to provide encouragement to schizophrenic patients aged 40, 50, or 60).
    - (Example 1) Identify eligible patients from those who will be seen on the same day, and list them by psychiatrists.
    - (Example 2) Identify the age of the patient and place a post-it on a paper chart showing the eligible patients.
    - (Example 3) The electronic medical record automatically notifies the psychiatrists that the patient is eligible for encouragement.
  - Decide on an easy-to-implement flow from the time the psychiatrists recognize eligible patients to the time the case managers offer encouragement.
    - (Example 1) The psychiatrist recommends to the patient that they should get an explanation of the cancer screening, and gives them a card; the encouragement is then offered to each patient with a card.
    - (Example 2) The psychiatrist instructs the nurse present in the counseling room to provide the encouragement.
  - Decide which staff members will be able to provide the encouragement.
    - Consider the number of eligible patients visiting the outpatient clinic per day.
    - Staff members who will implement the encouragement are expected to be nurses, mental health social workers, or psychologists. This encouragement method is feasible for staff who have mastered the basic skills of daily clinical practice in a psychiatric hospital.
  - Decide what will be recorded in the patient’s medical record when the encouragement is provided.
    - It is preferable to record the following information: i) whether education/navigation for cancer screening was provided, and ii) if it was deemed unnecessary, the reason (e.g., opportunity to receive screening at the workplace, or recent colonoscopy).
  - Identify patients who need follow-up contact and how to contact them next time.
    - (Example) Record in the patient’s medical record/appointment slip that they should be approached at the next visit.

*Two follow-up contacts are generally required, but this can be omitted for patients for whom it is deemed unnecessary. Most patients who indicated their willingness to undergo screening at the initial interview were able to complete the procedure without follow-up contact, and few changed their willingness to undergo screening as a result of follow-up co
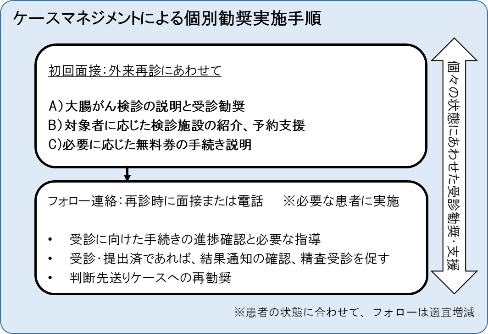
ntact.

- - Decide the time frame for providing the encouragement, on the basis of the cancer screening periods in the surrounding municipalities.
    - (Example) Provide the encouragement intensively in June–August.
  - The final decision about how to implement the encouragement will be made after listening to the opinions of the medical staff involved.
    - **
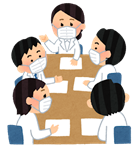
**Reflecting the opinions of those who will actually implement the recommendations and recognize it as their project is an important part of the process.

**Ongoing encouragement flyer in the following year**

- -
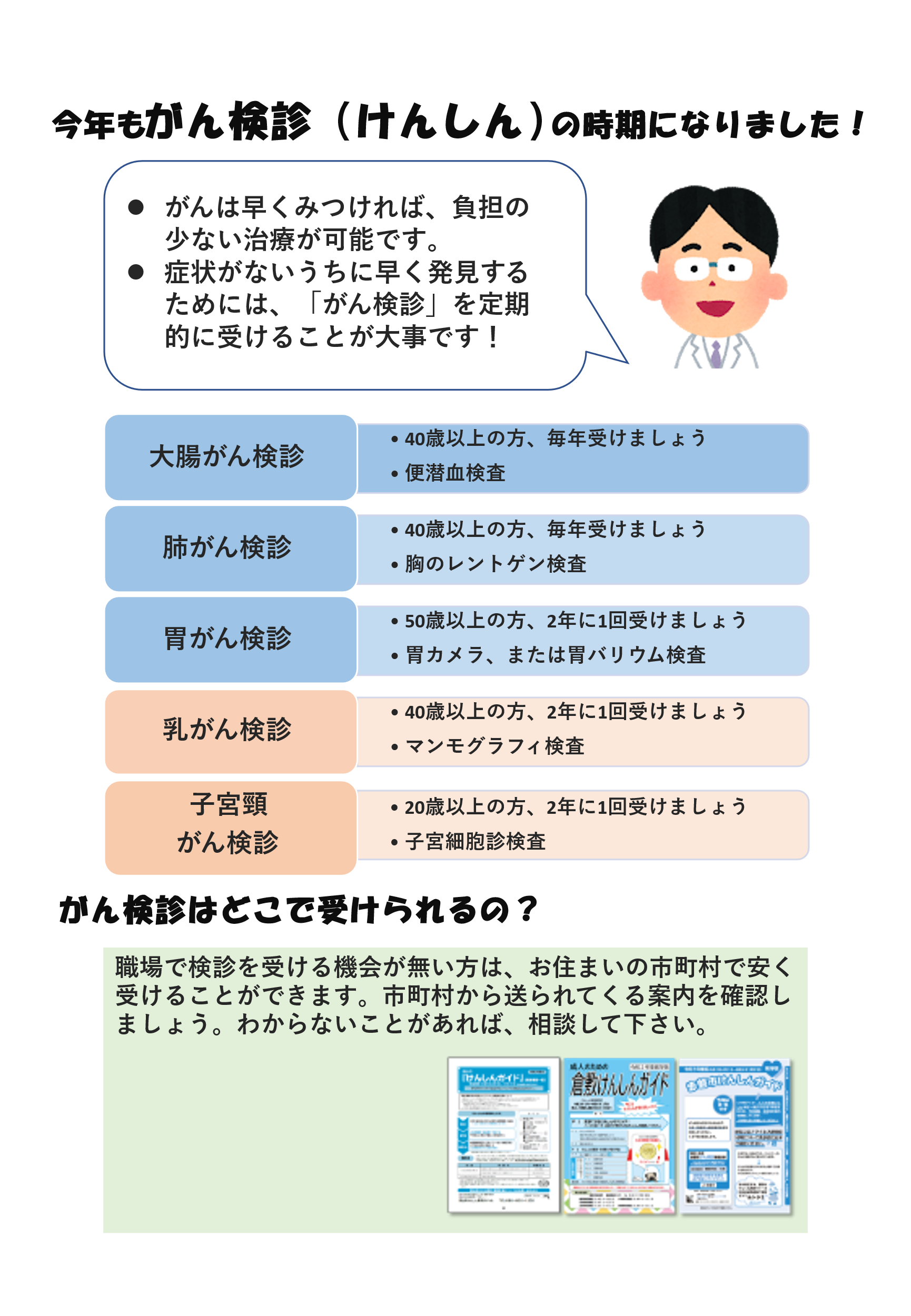
Patients who receive encouragement using case management will receive an encouragement flyer from their psychiatrists in the following year.
  - The encouragement flyer will be provided to outpatients with schizophrenia aged 40–69, the age group for which colorectal cancer screening is specifically recommended in Japan.
  - Modify the flyer to fit your facility and determine how it will be offered.

**３．Educating staff using the materials in this guide**

- ***3.1 Educate the medical staff about cancer screening and the methods of encouragement***
  - Education about cancer screening (Educational Resources 1 and 2)
  - Education about the methods of encouragement (Educational Resource 3)
- ***3.2*** ***Inform all outpatient staff about how to implement the encouragement***
  - Disseminate the operational procedures determined in Step 2 to outpatient psychiatrists, staff involved in the encouragement, and other staff.

**４．Team/on-site staff providing** **encouragement of cancer screening**

- - The team checks the operation and modifies it as necessary.
    - During the first 2 weeks after the start of the encouragement, the team will proactively check for any problems with the staff providing the encouragement. Corrected points will be shared with relevant staff.
  - At the end of the encouragement period, the team will interview the outpatient staff to understand the issues involved in the implementation of encouragement and make the best use of them in the next year.

**Educational Resources and Materials**

- Educational Resource 1 (general knowledge about cancer screening)
- Educational Resource 2 (knowledge about municipal cancer screening)
- Educational Resource 3 (methods of the encouraging cancer screening)
- Materials to be used in the encouragement using case management
- Encouragement flyer

**精神科医療機関における**

**がん検診勧奨実施ガイド**

令和3年度厚生労働科学研究

精神障害のある方に対するがん検診及びがん診療のアクセシビリティを向上するための実装研究

分担研究：かかりつけ精神科の臨床場面における精神障害者に対するがん検診勧奨法の

実施可能性の検討-多施設介入研究

2021年10月10日　第1.0版作成

**はじめに**

　進行がんで発見されながらも、手術や抗がん剤などによる標準的な治療を受けることができない統合失調症患者さんのケアを私達はしばしば経験します。がんの早期発見、早期治療にはがん検診が有効で、わが国では５つのがん検診を受診することが勧められており、市町村が、がん検診を案内しています。しかし、精神障害を抱える方は一般住民の約2分の1の受診率という格差があります。

　本ガイドに従い、かかりつけ精神科医療機関のスタッフによる個別のがん検診勧奨を行うと、最も受診率が低いことがわかっている統合失調症患者さんでも、一般住民と同程度の大腸がん検診受診率まで上昇します。がん検診が気になるけれど、よくわからないために受けない患者さんが多く、外来スタッフからの説明、案内がとても助かったという声をいただいています。

　精神障害を有する方のがん検診受診率、がん死亡率の格差を改善するため、皆様のご協力をよろしくお願いいたします。

主任研究者　藤原雅樹（岡山大学）

厚生労働科学研究班代表者　稲垣正俊（島根大学）

**以下の1～4のステップで、自施設で準備を整えてがん検診勧奨法を実施します**

**1.　施設長または部門長が、がん検診勧奨を運営する委員会を組織する**

**2.　運営委員会が、本ガイドの手順に従い、事前準備を行う**

**3.　本ガイドの資料を用いて、がん検診勧奨に関わるスタッフを教育する**

**4.　運営委員/スタッフが、がん検診勧奨を実施する**

**1．がん検診勧奨の実施を準備するチームを組織する**

- 多職種での運営委員会を構成します。外来部門にかかわる医師と、少なくとも看護師または/および精神保健福祉士によるチームが望ましいです。
- 運営委員会は、本ガイドの手順に従い事前準備を行い、運営の主体となります。
- 外来の仕組みを熟知している医師や看護師、相談員を含むことが望ましいです。
- がん検診勧奨運営委員会と人選について病院内に周知します。

**
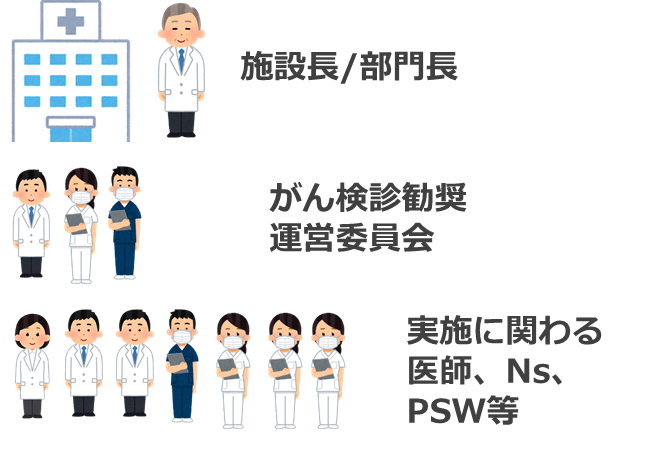
**

**２．運営委員会が、本ガイドの手順に従い、事前準備を行う**

- ***2.1　委員メンバー内で、がん検診、個別勧奨法への理解を深める***
  - 五大がん検診の内容について一般的な知識を理解する（教育資料①）。
  - 市町村がん検診の体系、受診の手続きを理解する（教育資料②）。
  - ケースマネジメントによる個別勧奨法のポイントを理解する（教育資料③）
- ***2.2　周辺市町村の「けんしんガイド」を確認し、勧奨資料を自施設に併せて修正する***
  - 「けんしんガイド」などの名称で、当該年度の5月下旬頃に各市町村のホームページで資料が公開されます。まずは、前年度のものを参考に準備を進めましょう。
- ***2.3　個別勧奨の運用方法を決める***
  - 診察する患者が、個別勧奨の対象（当該年度に40/50/60歳となる統合失調症患者へ実施することをまず目標とします）であることを担当医師が見逃さないための運用方法を決めます。
    - （案１）前日に翌日の再診患者リストから個別勧奨の対象者を同定して、担当医別にリストにして診察室に配る。
    - （案２）当日来院して紙カルテを出す際に年齢を確認し、個別勧奨の対象者カルテにポストイットを貼って医師に知らせる。
    - **
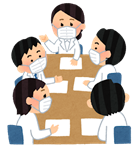
**（案３）電子カルテで自動的に個別勧奨の対象となる年齢の患者であることを担当医に知らせる。
  - 担当医師が個別勧奨の対象者であると気づいてから、資料を用いてがん検診勧奨を行うまでの、実施しやすい流れを決めます。
    - （案１）主治医がカードを渡して説明し、患者はカードを診察後に個別勧奨を行うスタッフ/受付に渡す。
    - （案２）外来についている看護師に、個別勧奨を依頼する。
  - 個別勧奨を実施できるようになってもらうスタッフの範囲を検討します。
    - 勧奨対象患者が、一日におよそ何人受診するかを調べて判断材料にします。
    - 実施するスタッフは、看護師、精神保健福祉士、心理士を想定しています。日常臨床の基本的な相談・支援業務を習得していれば、実施可能な勧奨法です。
  - 個別勧奨を行った際に、カルテへ記録する内容について確認します。
    - 次の①、②が区別できるように記載します：①資料を用いて勧奨した患者、②声をかけたが資料を用いた勧奨は不要と判断した患者（職場で受ける、最近大腸カメラを受けた、等）
  - フォロー連絡が必要な患者の把握、次回に勧奨する方法を検討します。
    - （案）次回再診時に声をかけるよう、カルテ/予約票などに記録し、担当医または勧奨にかかわるスタッフが再診時に声掛けを行う


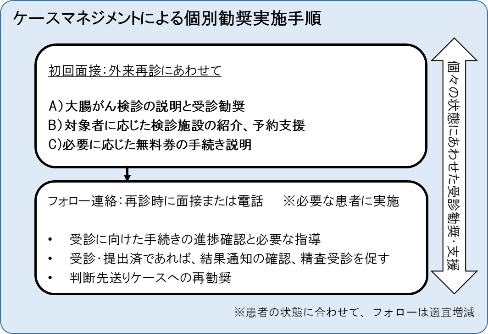


※再診時に2回フォロー連絡を行うことを基本としていますが、フォロー連絡は不要と判断したケースでは省略可能です。初回面接で受ける意思を示した人の多くはフォロー連絡がなくても受診手続きができており、また、フォロー連絡で受診意思が変わった方はほとんどいませんでした。

- - 勧奨を行う期間について、周辺市町村のがん検診期間を踏まえて決定します。
    - （例）6月-8月で集中的に実施する。
  - 運用方法について、実施にかかわるスタッフの意見を聞いて最終的に決定します。
    - 実際に勧奨を実施する方々の意見を反映し、自分たちの取り組みとして認識してもらうことは、大切なプロセスです。


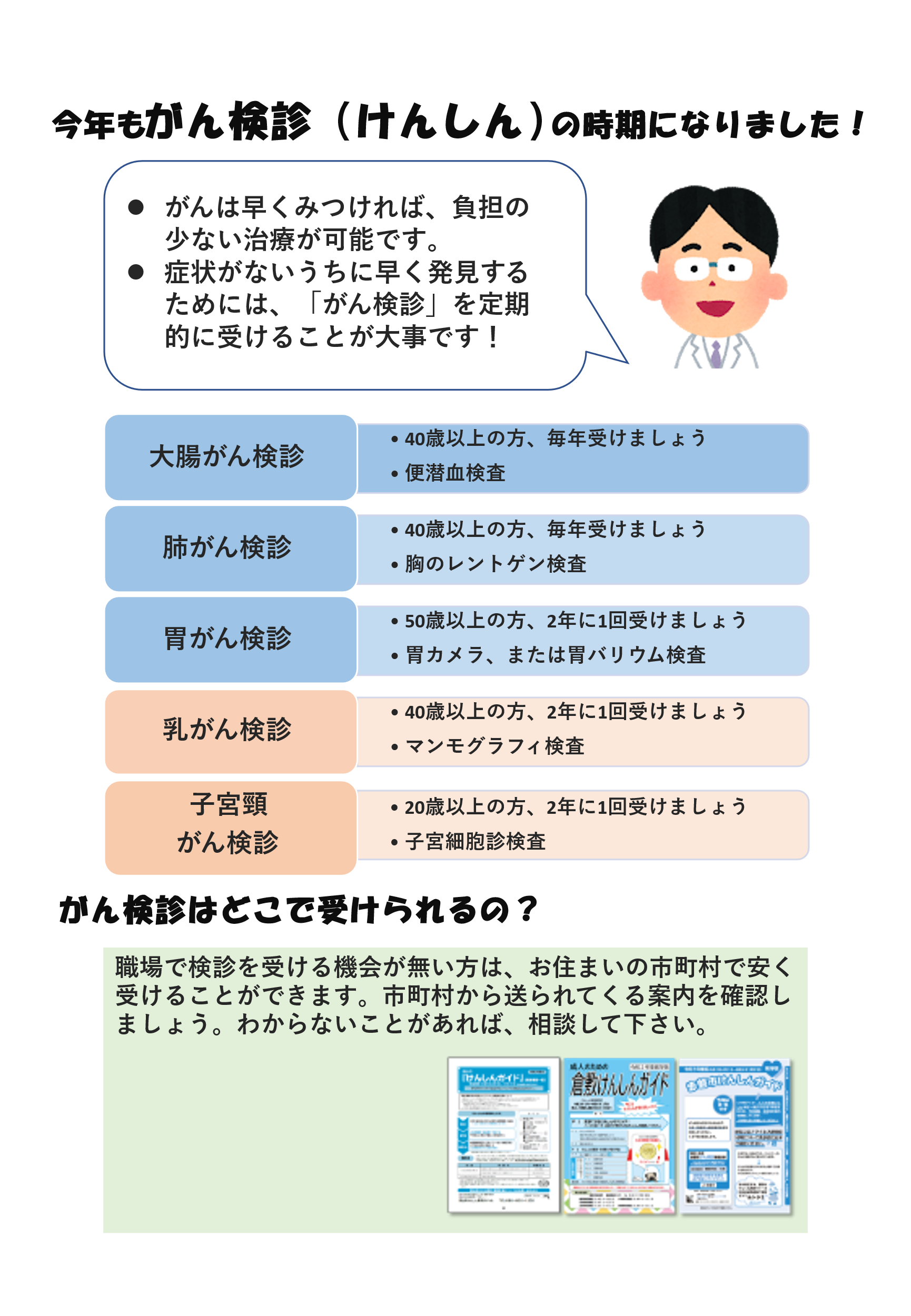
***【翌年度に実施する継続的なチラシ勧奨】***

- *ケースマネジメントによる個別勧奨を受けた翌年度以降は、担当医が再診のタイミング等を利用して、チラシを用いてがん検診について声掛けを継続します（支援が必要な場合は適宜サポートします）。*
- *個別勧奨を受けていない患者さんにチラシ勧奨を行っても不利益はないことから、大腸がん検診を特にお勧めす*る年齢である40-69歳の統合失調症外来患者さん全員に対して勧奨します。
  - 自施設にあわせてチラシを修正し、運用方法を決めます。

**３．本ガイドの資料を用いて、がん検診勧奨に関わるスタッフを教育する**

- ***3.1　個別勧奨の実施に関わるスタッフに、がん検診および勧奨法の教育を行う***
  - がん検診について教育を行います（教育資料①、②）。
  - 勧奨法について教育を行います（教育資料③）。
- ***3.2*外来でのがん検診勧奨の運用方法について、外来スタッフに周知する**
  - 2で決めた運用を、外来医師、勧奨に関わるスタッフ、その他のスタッフまで広く周知します。

**４．運営委員/スタッフが、がん検診勧奨を実施する**

- 運営委員会は、がん検診勧奨の運用状況について適宜確認し、修正、改善点があれば対応します。
  - 開始直後の１～２週間は、勧奨実施スタッフに問題点がないか積極的に確認します。修正点があれば対応し、皆へ共有するようにします。
- 運営委員会は、がん検診勧奨期間の終了後、外来医師や勧奨実施スタッフにアンケート等を行い、実施した勧奨の確認、課題を把握して、次年度に生かします。

**【資料】**

- 教育資料①（がん検診について　総論）
- 教育資料②（市町村検診について）
- 教育資料③（勧奨法について）
- 個別勧奨資料
- 勧奨チラシ
