# Supplementary material for: A feasibility study of provider-level implementation strategies to improve access to colorectal cancer screening for patients with schizophrenia: ACCESS2 (N-EQUITY 2104) trial
Source: Implement Sci Commun. 2024 Jan 4;5:2. doi: 10.1186/s43058-023-00541-0 (PMC10768128; doi:10.1186/s43058-023-00541-0)
Supplement: Supplementary file 2 — Additional file 2: Supporting Table 1. Intervention using case management to encourage CRC screening. [file 43058_2023_541_MOESM2_ESM.docx]

**Supporting Table 1. Intervention using case management to encourage CRC screening**

| **Elements of the intervention** | **TIDieR checklist explanation** | | | | | |
| --- | --- | --- | --- | --- | --- | --- |
|  | **Why** | **What** | **Who provided** | **How** | **Where** | **When and how much** |
| Education for CRC screening | Fujiwara et al. 2019, 2021 and Yamada et al. 2022 | Patients are educated about cancer screening using pamphlets according to their function. The pamphlet includes the following information: i) cancer risk, ii) importance of early detection by cancer screening, iii) recommendation to undergo colorectal cancer screening, iv) information about how to proceed with colorectal cancer screening. | Case mangers (nurses or mental health social workers) | In-person counseling | Outpatient clinics in psychiatric hospital | This element is conducted in the first session (average 13 minutes) |
| Assistance with making decisions and an appointment for CRC screening | Same as above | Patients will decide with staff whether or not to undergo CRC screening during the current year. If they do, they will discuss where and when they will be screened and receive assistance with the process. | Same as above | Same as above | Same as above | Same as above |
| Assistance with obtaining a coupon for free screening | Same as above | Patients receive assistance in obtaining free coupons as needed. | Same as above | Same as above | Same as above | Same as above |
| Follow-up | Same as above | Patients are asked about the progress of the procedure to undergo CRC screening. Patients receive support or encouragement, depending on their situation. | Same as above | In-person or telephone counselling | Same as above | Follow-up sessions (approximately 3 minutes) are generally held twice, but may be skipped on the basis of clinical judgement |

TIDieR: Template for Intervention Description and Replication; CRC: colorectal cancer
